# Dental professionals’ knowledge and awareness of teledentistry

**DOI:** 10.1186/s12903-026-08717-5

**Published:** 2026-06-03

**Authors:** Abla Arafa, Nour AbdelShakoor, Amira Hegazy, Mohamed Salah, Malak Abdelazim, Ahmed Ezzat, Moustafa Moufeed, Mohamed Youssef, Clara Raymond, Fares Jamal

**Affiliations:** 1 Department of Pediatric Dentistry and Dental Public Health, Faculty of Oral and Dental Medicine, Misr International University, 28 Cairo-El Ismailia Road, Cairo, Egypt; 2 Dental student, Faculty of Oral and Dental Medicine, Misr International University, Cairo, Egypt

**Keywords:** Teledentistry, Knowledge assessment, Awareness, Dental Professionals, Educational intervention

## Abstract

**Purpose:**

To assess teledentistry (TD) knowledge and enhance its awareness among different categories of dental professionals, interns and dental students.

**Methods:**

This cross-sectional survey used self-administered prevalidated pretested questionnaire (electronic version) that is divided into two sections; the first section focused on participants’ demographic data, and the second section assessed knowledge, attitude, practice perspectives, and perceived barriers towards TD. Re-assessment of TD knowledge was conducted following educational intervention. Kruskal-Wallis test was used to assess for significance at *p* < 0.05 using SPSS (v. 25).

**Results:**

A total of 553 responses were collected from dental students (*N* = 241), interns (*N* = 96), and dentists (*N* = 216). The baseline awareness was significantly low (27.7%), particularly among interns and dentists (*p* < 0.001), with projection against its usefulness in dental practice (*p* < 0.001). Students presented positive attitude towards TD time and costs reduction (*p* < 0.001). Students agreed on necessity of patients’ consents and replacing traditional dental setting with digital practice (*p* < 0.001). For perceived barriers, patients’ low literacy and charging fees were the prime barriers against TD integration. Enhanced teledentistry awareness following educational intervention was significant among students, followed by dentists, and interns.

**Conclusions:**

Teledentistry baseline awareness among dental professionals was limited, particularly dentists and interns. Patient’s privacy, cost-effectiveness and practical applicability of teledentistry were among the main concerns particularly expressed by the dentist. Educational intervention positively enhanced teledentistry awareness and would encourage its use for upskilling health care workers, diagnosis and management of oral diseases, highlighting the value of structured teaching.

**Supplementary Information:**

The online version contains supplementary material available at 10.1186/s12903-026-08717-5.

## Introduction

Teledentistry (TD) is a novel approach in modern dental healthcare that applies telecommunications technology to deliver oral health services, consultations, and instruction in a remote manner. According to the American Dental Association, TD can be defined as the utilization of electronic information, imaging, and communication technologies to provide dental care delivery, diagnosis, consultation, treatment, transfer of dental information, and education [1]. It is derived from the science of tele-health, which has shown a significant effect on the healthcare profession and can be applied widely to masses of populations where utilization of dental care was described as poor or underserved. Persistence of oral health inequalities remained overtime particularly among marginalized people [2]. With the COVID-19 Pandemic, along with all other health sectors, dental practice was also affected. The pandemic proved that there is a need to restructure and introduce innovative electronic methods of healthcare delivery to pursue dental care with minimal risk of disease transmission [3].

Despite that oral health care remain mainly offered via traditional dental services, TD may provide an alternative support in diagnosis, patient monitoring and establishment of treatment planning [4]. Teledentistry transcends geographical barriers, enabling dental professionals to connect with patients virtually, thereby expanding access to care and improving overall oral health outcomes. As technology continues to advance, TD has developed as a solution to address discrepancies in dental care access, particularly in underserved or rural communities where access to traditional dental clinics is considered limited [5]. The concept of TD incorporates various modalities, including live video conferencing for real-time consultations, store-and-forward technology for asynchronous communication of patient data and images, remote monitoring of oral health conditions, and mobile health applications (m-health) that facilitate patient engagement and education [6]. These technologies not only enhance conveniences for patients but also empower dental providers to deliver timely interventions, preventive care, and treatment planning distantly [7]. Furthermore, TD aligns with the evolving preferences of patients who seek efficient and cost-effective healthcare solutions. It promotes proactive dental care management, early detection of oral health issues, and continuity of care, thereby potentially reducing emergency visits and improving overall patient satisfaction. However, despite its benefits, TD is also opposed by challenges such as ensuring data security, maintaining regulatory compliance, and overcoming technological barriers, particularly in remote settings [8, 9].

The adoption of TD continues to grow, as it promises to reshape the landscape of dental care delivery, making oral health services more accessible and responsive to the diverse needs of patients globally, particularly with the rising advancement in artificial intelligence and telecommunication technologies that could encourage the TD implementation into regular practice to facilitate accessibility and polish the efficacy of oral health care services [10, 11]. Yet, the lack of awareness about the innovative use of TD among the different categories of students and dental professionals could hinder the spread of TD use in different dental fields [12].

The current research gap of knowledge and population arises from the scanty studies explored the knowledge and perception of different categories of dental professionals regarding the Teledentistry clinical implementation in the dental practice. Therefore, the aim of this study is to assess the knowledge and perception of Teledentistry clinical application and the barriers to practical implementations among dental students, interns, and dentists, compared to enhanced awareness following the introduction of educational intervention.

## Methods

### Ethical consideration

The ethical approval to conduct this study was obtained from Institutional Ethical Research Committee. A brief statement describing the study was inserted at the beginning of the electronic survey with clarification that participation in the study is totally voluntary. By accepting to respond to the administered questionnaire and continuing the survey, the participants consisted to be part of the study.

### Sample size calculation

A convenient sampling method was used in this study. A pilot investigation was conducted on 60 participants, whose responses were not included in the final analysis. Based on the pilot findings, only 30% of participants demonstrated prior awareness of Teledentistry. A baseline TD knowledge level of 50% among dental professionals [13], with an assumed 20% difference in the target group were used for sample size calculation using ClinCalc sample size calculator. To achieve 80% study power at a two-sided α level of 0.05, a minimum sample size of 194 participants was required.

### Study design

This study employed a quasi-experimental pre-test/post-test design to assess the level of knowledge and to evaluate the effect of educational intervention on responders’’ knowledge and awareness of Teledentistry. A self-administered, structured electronic prevalidated and pretested questionnaire was adopted for assessing knowledge and perceptions of undergraduate bachelor degree dental students, interns and dentists towards TD [10]. The questionnaire consisted of two major sections; the first section focused on the demographic characteristics of the participants (six items), including age, gender, level of education, designation, hours of internet usage per day, area, and years of dental practice. The second section inquired responses about the domains of knowledge (seven items), attitudes (seven items), practices (seven items), and perceived barriers towards applications and usage of TD (six items), particularly within the local context to identify responses that reflect the national dental healthcare settings. The questionnaire was tested for internal consistency using Cronbach’s alpha, with α = 0.789, indicating acceptable reliability. The questionnaire validity was tested using item-level (I-CVI: ranged 0.80-1.00), and scale-level (S-CVI = 0.92), indicating excellent validity.

The questionnaire contained both dichotomous (Yes/No) closed-ended questions and items measured on a five-point Likert scale ranging from “strongly agree”, to “strongly disagree”, to allow assessment of participants’ level of agreement across different domains. The selected questionnaire was composed in simple, readable language to facilitate understanding. A statement highlighting the study was inserted at the outset to describe study objectives and encourage the response rate. The questionnaire was short and partitioned with logical sections to limit respondent fatigue.

### Educational intervention

To enhance Teledentistry knowledge and awareness among the participants, a 10-minute educational intervention was administered after completing the baseline questionnaire. The focus of this concise, structured presentation was based on current evidence to clarify the basic knowledge regarding the definition, principles, applications, benefits, and limitations of teledentistry relevance to dental practice. Additionally, an explanation was provided on teledentistry applications for remote consultations, patient diagnosis, follow-up standards, referral, and interdisciplinary oral health care delivery, particularly in underserved areas with special ethical consideration as regards patients’ confidentiality. The educational material was revised by a professor of Dental Public Health to ensure clarity and comprehensiveness. The educational intervention was provided to all responders via the same survey link, followed by re-testing the knowledge section to assess improvement in responders’ awareness. The awareness was assessed using a single direct item asking of TD awareness, where responses were used to categorize participants as either aware or not aware about TD. The attitude, practice, and perceived barriers domains were not re-assessed, as they require behavioral adaptation over an extended time to reach reliable re-assessment [14].

The survey was disseminated electronically to both current dental students, interns through email and learning management systems. An electronic (Google Forms) version of the self-administered questionnaire was developed using an online survey platform and distributed to dentists attending the Egyptian Dental Syndicate during the period from December 2024 to February 2025. To ensure consistency, standardized procedures using the same survey instrument, and instructions were followed throughout data collection period to minimize variability. In addition, to enhance outreach and ensure broader exposure to the target population, the questionnaire link was disseminated via professional WhatsApp groups. The invitation encompassed a detailed outline of the study’s objectives, a concise overview of TD along with its prospective advantages in dental practice, and a solicitation for voluntary involvement. Participants had to give their voluntary consent before accessing the questionnaire. To ensure maximum response rate and minimum selection bias, participants were informed that their responses would remain anonymous and confidential. The implementation of an online survey enhanced accessibility, and secured data acquisition, given that responses were recorded electronically without the capture of any personally identifiable information. Participants were permitted to respond only once to the survey.

### Statistical analysis

The responses were statistically analyzed using SPSS software, Version 25.0 (SPSS Inc., Chicago, IL, USA) to test for statistical significance at *p* < 0.05. Only complete responses were included for statistical analysis. The Chi-square test was used to assess demographic data among the study groups. The 5-point Likert scale responses were tested among dental students, interns, and dentists using the Kruskal-Wallis test, followed by Dunn’s post-hoc test for multiple comparisons. McNemar’s test was used to assess changes in TD awareness following the educational intervention. The calculation of the effect size was carried using the following equation:

The responses were statistically analyzed using SPSS software, Version 25.0 (SPSS Inc., Chicago, IL, USA) to test for statistical significance at *p* < 0.05. Only complete responses were included for statistical analysis. The Chi-square test was used to assess demographic data among the study groups. The 5-point Likert scale responses were tested among dental students, interns, and dentists using the Kruskal-Wallis test, followed by Dunn’s post-hoc test for multiple comparisons. McNemar’s test was used to assess changes in TD awareness following the educational intervention. The calculation of the effect size was carried using the following equation: 

$$\eta^{2}=\frac{H-k+1}{N-k}$$

Where H = Kruskal–Wallis test statistic, k = number of groups, N = total sample size.

## Results

A total of 553 complete responses were received from bachelor degree undergraduate dental students (*N* = 241), dental interns (*N* = 96), and dentists (*N* = 216). Among a total of 2585 survey invitations sent via email and professional dental WhatsApp groups, 553 participants completed the questionnaire, giving a response rate of 21.39%. The demographic characteristics of the participants are shown in Tables 1 and 2. Assessment of age group distribution revealed that responses from older age group (above 40-yr-old) were predominantly received from dentists, while the younger age group (below 24-yr-old) was mainly represented by undergraduate students and interns (*p* < 0.001). Genders distribution showed statistically insignificant difference among the groups (*p* > 0.05). Among participants reporting high daily use of the internet (more than 6-hour per day), undergraduates contributed the largest proportion, while dentists were more frequently represented in the category of intermediate usage (from 3- to 6-hour per day). The higher clinical experience (more than 10-years) was reported significantly by female dentists (*p* = 0.003) regardless their specialty (*p* = 0.168).

**Table 1:** Age group distribution and internet usage characteristics of the study participants

Variables		BDS *N* = 241 (%)	Interns *N* = 96 (%)	Dentists *N* = 216 (%)	Total *N* = 553 (%)	*p*-value
Age	< 24	239 (43.2)	96 (17.4)	12 (2.2)	347 (62.7)	0.001
	25–40	2 (0.4)	0 (0.0)	98 (17.7)	100 (18.1)	
	> 40	0 (0.0)	0 (0.0)	106 (19.2)	106 (19.2)	
Internet usage (hr/d)	3>	14 (2.5)	9 (1.6)	20 (3.6)	43 (7.8)	0.004
	3–6	147 (26.6)	66 (11.9)	157 (28.4)	370 (66.9)	
	6<	80 (14.5)	21 (3.8)	39 (7.1)	140 (25.3)	

*BDS* Bachelor degree undergraduate student

**Table 2:** Designation and gender distribution characteristics of the study participants

Variables			*N*=553	Gender *N*(%)		*p*-value	
				Female 302 (54.6%)	Male 251 (45.4%)		
Designation	BDS		241 (43.6)	132 (23.9)	109 (19.7)		0.66
	Academic level	1^st^ yr	20 (8.3)	10 (4.1)	10 (4.1)	0.404	
		2^nd^ yr	32 (13.3)	16 (6.6)	16 (6.6)		
		3^rd^ yr	71 (29.5)	44 (18.3)	27 (11.2)		
		4^th^ yr	54 (22.4)	32 (13.3)	22 (9.1)		
		5^th^ yr	64 (26.6)	30 (12.4)	34 (14.1)		
	Intern		96 (17.4)	56 (10.1)	40 (7.2)		
	Dentist		216 (39.1)	114 (20.6)	102 (18.4)		
Dentist	Experience	5 yr>	69 (31.9)	30 (13.9)	39 (18.1)	0.003	
		5-10 yr	73 (33.8)	33 (15.3)	40 (18.5)		
		10 yr<	74 (34.3)	51 (23.6)	23 (10.6)		
	Specialty	General	168 (77.8)	91 (42.1)	77 (35.6)	0.168	
		Restorative	14 (6.5)	4 (1.9)	10 (4.6)		
		Surgery	21 (9.7)	10 (4.6)	11 (5.1)		
		Preventive	13 (6.0)	9 (4.2)	4 (1.9)		

*BDS*  Bachelor degree undergraduate students

The results of assessment of TD knowledge, attitude, practice and barriers among the participants are presented in Table 3. Awareness of TD was found significantly low (27.7%), particularly among the dental interns and dentists (*p* < 0.001). Most of the interns were neutral regarding the usefulness of TD in different dental branches (H = 23.153, *p* < 0.001, partial η² = 0.038, reflecting a small effect size), disagree on TD applicability in current dental practice (H = 78.646, *p* < 0.001, partial η² = 0.136, reflecting a large effect size), and consulting patients’ problems effectively (H = 16.273, *p* < 0.001, partial η² = 0.022, reflecting a small effect size). The responses of dentists towards possibility of TD to have positive impact on oral health care access improvement revealed neutral or disagreement, while students’ and interns’ responses were towards agreement (H = 43.987, *p* < 0.001, partial η² = 0.051, reflecting a small effect size). The undergraduate students were more positive towards the effectiveness of TD in education and upskilling health care workers (H = 16.273, *p* < 0.001, partial η² = 0.073, reflecting a medium effect size). Both groups of students and dentists showed responses leaning more towards agreement as regards the possible usefulness of TD in diagnosis and management planning of oral diseases (H = 11.913, *p* < 0.001, partial η² = 0.014, reflecting a small effect size).

**Table 3:** Participants’ responses regarding their knowledge, Attitude, Practice and Barrier towards Teledentistry

Variables	BDS *N* = 241 (%)	Interns *N* = 96 (%)	Dentists *N* = 216 (%)	Total *N* = 553 (%)	H	*p*-value
A. Knowledge						
Q1: Are you aware of Teledentistry?						
Yes	87 (15.7)	18 (3.3)	48 (8.7)	153(27.7)		0.001
No	154 (27.8)	78 (14.1)	168 (30.4)	400(72.3)		
Q2: Teledentistry is useful in all branches of dentistry?						
SA	34 (6.1)	0 (0.0)	29 (5.2)	63 (11.4)	23.153	0.001
A	79 (14.3)	13 (2.4)	50 (9.0)	142 (25.7)		
N	101 (18.3)	72 (13.0)	108 (19.5)	281 (50.8)		
D	27 (4.9)	11 (2.0)	29 (5.2)	67 (12.1)		
SD	0 (0.0)	0 (0.0)	0 (0.0)	0 (0.0)		
	Interns-dentists: *p* = 0.003, Interns-BDS: *p* < 0.001, Dentists-BDS: *p* = 0.167					
Q3: Could current dental practices be accomplished through teledentistry?						
SA	9 (1.6)	0 (0.0)	12 (2.2)	21 (3.8)	78.646	0.001
A	72 (13.0)	3 (0.5)	46 (8.3)	121 (21.8)		
N	100 (18.1)	17 (3.1)	84 (15.2)	201 (36.4)		
D	58 (10.5)	76 (13.7)	68 (12.3)	202 (36.5)		
SD	2 (0.4)	0 (0.0)	6 (1.1)	8 (1.5)		
	Interns-dentists: *p* < 0.001, Interns-BDS: *p* < 0.001, Dentists-BDS: *p* = 0.093					
Q4: Can oral health care access be improved by teledentistry?						
SA	36 (6.5)	10 (1.8)	26 (4.7)	72 (13.0)	31.843	0.001
A	142 (25.7)	63 (11.4)	75 (13.6)	280 (50.7)		
N	47 (8.5)	18 (3.3)	78 (14.1)	143 (25.9)		
D	16 (2.9)	5 (0.9)	37 (6.7)	58 (10.5)		
SD	0 (0.0)	0 (0.0)	0 (0.0)	0 (0.0)		
	Dentists-interns: *p* < 0.001, Dentists-BDS: *p* < 0.001, Interns-BDS: *p* = 0.915					
Q5: Can teledentistry be useful for dental education and for upskilling health care workers through the internet?						
SA	59 (10.7)	13 (2.4)	11 (2.0)	83 (15.1)	43.987	0.001
A	111 (20.1)	52 (9.4)	95 (17.2)	258 (46.7)		
N	49 (8.9)	16 (2.9)	51 (9.2)	116 (21)		
D	12 (2.2)	10 (1.8)	32 (5.8)	54 (9.8)		
SD	10 (1.8)	5 (0.9)	27 (4.9)	42 (7.6)		
	Dentists-interns: *p* < 0.001, Dentists-BDS: *p* < 0.001, Interns-BDS: *p* = 0.098					
Q6: Can teledentistry be useful in diagnosis and management of oral diseases?						
SA	33 (6.0)	5 (0.9)	17 (3.1)	55 (10.0)	11.913	0.003
A	94 (17.0)	28 (5.1)	105 (19.0)	227 (41.1)		
N	60 (10.8)	36 (6.5)	58 (10.5)	154 (27.8)		
D	46 (8.3)	20 (3.6)	25 (4.5)	91 (16.4)		
SD	8 (1.4)	7 (1.3)	11 (2.0)	26 (4.7)		
	Interns-dentists: *p* < 0.001, Interns-BDS: *p* < 0.001, Dentists-BDS: *p* = 0.726					
Q7: Would teledentistry be helpful in consulting an expert about a patient’s problem effectively?						
SA	32 (5.8)	6 (1.1)	30 (5.4)	68 (12.3)	16.273	0.001
A	50 (9.0)	14 (2.5)	43 (7.8)	107 (19.3)		
N	58 (10.5)	22 (4.0)	84 (15.2)	164 (29.5)		
D	62 (11.2)	41 (7.4)	33 (6.0)	136 (25.2)		
SD	39 (7.1)	13 (2.4)	26 (4.7)	78 (14.2)		
	Interns-dentists: *p* < 0.001, Interns-BDS: *p* < 0.001, Dentists-BDS: *p* = 0.846					
B. Attitude						
Q8: Do you feel Teledentistry can monitor the patient’s condition?						
SA	18 (3.3)	6 (1.1)	37 (6.7)	61 (11.1)	11.452	0.003
A	70 (12.7)	20 (3.6)	51 (9.2)	141 (25.5)		
N	87 (15.7)	32 (5.8)	79 (14.3)	198 (30.8)		
D	40 (7.2)	23 (4.2)	29 (5.2)	92 (16.6)		
SD	26 (4.7)	15 (2.7)	20 (3.6)	61 (11.0)		
	Interns-dentists: *p* < 0.001, Interns-BDS: *p* = 0.038, Dentists-BDS: *p* = 0.085					
Q9: Do you feel teledentistry could be a way of oral health care delivery?						
SA	29 (5.2)	20 (3.6)	12 (2.2)	61 (11.0)	46.321	0.001
A	100 (18.1)	22 (4.0)	42 (7.6)	164 (29.7)		
N	70 (12.7)	31 (5.6)	76 (13.7)	177 (32.0)		
D	38 (6.9)	14 (2.5)	59 (10.7)	111 (20.1)		
SD	4 (0.7)	9 (1.6)	27 (4.9)	40 (7.2)		
	Dentists-interns: *p* < 0.001, Dentists-BDS: *p* < 0.001, Interns-BDS: *p* = 0.237					
Q10: Do you feel teledentistry could make dental examination easier**?**						
SA	35 (6.3)	5 (0.9)	17 (3.1)	57 (10.3)	10.037	0.007
A	67 (12.1)	23 (4.2)	53 (6.9)	143 (23.2)		
N	89 (16.1)	38 (6.9)	87 (15.7)	214 (38.7)		
D	34 (6.1)	18 (3.3)	53 (6.9)	105 (16.3)		
SD	16 (2.9)	12 (2.2)	6 (1.1)	34 (6.2)		
	Interns-BDS: *p* = 0.011, Interns-dentists: *p* = 0.757, Dentists-BDS: *p* = 0.075					
Q11: Do you think that teledentistry saves time for the dentist**?**						
SA	66 (11.9)	5 (0.9)	17 (3.1)	88 (15.9)	87.543	0.001
A	114 (20.6)	41 (7.4)	56 (10.1)	211 (31.1)		
N	51 (9.2)	33 (6.0)	106 (19.2)	190 (34.4)		
D	10 (1.8)	17 (3.1)	37 (6.7)	64 (11.6)		
SD	0 (0.0)	0 (0.0)	0 (0.0)	0 (0.0)		
	Dentists-BDS: *p* < 0.001, Interns-BDS: *p* < 0.001, Dentists-interns: *p* = 0.204					
Q12: Do you feel teledentistry could reduce the cost of dental services?						
SA	25 (4.5)	6 (1.1)	12 (2.2)	43 (7.8)	46.465	0.001
A	96 (17.4)	10 (1.8)	52 (9.4)	158 (28.6)		
N	77 (13.9)	35 (6.3)	97 (17.5)	209 (31.7)		
D	41 (7.4)	33 (6.0)	36 (6.5)	110 (19.9)		
SD	2 (0.4)	12 (2.2)	19 (3.4)	33 (6.0)		
	Interns- dentists: *p* = 0.004, Interns-BDS: *p* < 0.001, Dentists-BDS: *p* < 0.001					
Q13: Do you feel videoconference could be used to educate dental students effectively?						
SA	48 (8.7)	29 (5.2)	39 (7.1)	116 (21.0)	12.172	0.002
A	103 (18.6)	45 (8.1)	89 (16.1)	237 (42.8)		
N	66 (11.9)	18 (3.3)	61 (11.0)	145 (26.2)		
D	20 (3.6)	4 (0.7)	27 (4.9)	51 (9.2)		
SD	4 (0.7)	0 (0.0)	0 (0.0)	4 (0.7)		
	Dentists-interns: *p* < 0.001, BDS-interns: *p* = 0.004, Dentists-BDS: *p* = 0.404					
Q14: Do you feel teledentistry could improve accessibility of dental specialists to distant communities for their oral needs?						
SA	51 (9.2)	14 (2.5)	37 (6.7)	102 (8.4)	0.589	0.745
A	99 (17.9)	57 (10.3)	112 (20.3)	268 (48.4)		
N	66 (11.9)	18 (3.3)	34 (6.1)	118 (21.3)		
D	25 (4.5)	7 (1.3)	33 (6.0)	65 (11.8)		
SD	0 (0.0)	0 (0.0)	0 (0.0)	0 (0.0)		
C. Practice						
Q15: Do you feel patients would provide consent to share their dental reports through teledentistry to another dentist?						
SA	39 (7.1)	20 (3.6)	32 (5.8)	91 (16.5)	29.397	0.004
A	126 (22.8)	40 (7.2)	55 (9.9)	221 (39.9)		
N	56 (10.1)	22 (4.0)	77 (13.9)	155 (28.0)		
D	18 (3.3)	14 (2.5)	44 (8.0)	76 (5.8)		
SD	2 (0.4)	0 (0.0)	8 (1.4)	10 (1.8)		
	Dentists-interns: *p* = 0.001, Dentists-BDS: *p* < 0.001, Interns-BDS: *p* = 0.554					
Q16: Do you feel usage of teledentistry would support an initiative for delivering oral health to everyone on a national level?						
SA	47 (8.5)	14 (2.5)	11 (2.0)	72 (13.0)	6.611	0.037
A	86 (15.6)	30 (5.4)	94 (17.0)	210 (38.0)		
N	66 (11.9)	32 (5.8)	70 (12.7)	168 (25.4)		
D	26 (7.4)	20 (3.6)	32 (5.8)	78 (16.8)		
SD	16 (2.9)	0 (0.0)	9 (1.6)	24 (4.3)		
	Dentists-interns: *p* = 0.670, Dentists-BDS: *p* = 0.013, Interns-BDS: *p* = 0.133					
Q17: Do you think that dental examinations are accurate via computers and intraoral camera as in the traditional office setting?						
SA	39 (7.1)	8 (1.4)	12 (2.2)	59 (8.7)	0.823	0.663
A	47 (8.5)	25 (4.5)	53 (9.6)	125 (22.6)		
N	68 (12.3)	31 (5.6)	86 (15.6)	185 (33.5)		
D	65 (11.8)	22 (4.0)	36 (6.5)	123 (22.3)		
SD	22 (4.0)	10 (1.8)	29 (5.2)	61 (11.0)		
Q18: Does teledentistry include usage of smartphone applications for patient consultation?						
SA	39 (7.1)	10 (1.8)	11 (2.0)	60 (10.9)	18.565	0.001
A	90 (16.3)	40 (7.2)	61 (11.0)	191 (34.5)		
N	73 (13.2)	27 (4.9)	99 (17.9)	199 (36.0)		
D	22 (4.0)	12 (2.2)	25 (4.5)	59 (10.7)		
SD	17 (3.1)	7 (1.3)	20 (3.6)	44 (8.0)		
	Dentists-interns: *p* = 0.015, Dentists-BDS: *p* < 0.001, Interns-BDS: *p* = 0.420					
Q19: Do you feel teledentistry is easy to use?						
SA	33 (6.0)	9 (1.6)	12 (2.2)	54 (9.8)	12.958	0.002
A	88 (15.9)	42 (7.6)	72 (13.0)	202 (36.5)		
N	94 (17.0)	26 (4.7)	86 (15.6)	206 (37.3)		
D	22 (4.0)	13 (2.4)	30 (5.4)	65 (11.8)		
SD	4 (0.7)	6 (1.1)	16 (2.9)	26 (4.7)		
	Dentists-interns: *p* = 0.049, Dentists-BDS: *p* < 0.001, Interns-BDS: *p* = 0.448					
Q20: Do you feel hands-on training is required in using teledentistry?						
SA	61 (11.0)	24 (4.3)	62 (11.2)	147 (26.5)	2.083	0.353
A	112 (20.3)	48 (8.7)	106 (19.2)	266 (48.2)		
N	54 (9.8)	19 (3.4)	40 (7.2)	113 (21.4)		
D	14 (2.5)	5 (0.9)	8 (1.4)	27 (4.8)		
SD	0 (0.0)	0 (0.0)	0 (0.0)	0 (0.0)		
Q21: Do you feel a lecture/course is required to learn about teledentistry?						
SA	72 (13.0)	24 (4.3)	70 (12.7)	166 (30.0)	8.910	0.012
A	108 (19.5)	49 (8.9)	122 (22.1)	279 (50.5)		
N	47 (8.5)	15 (2.7)	24 (4.3)	83 (15.5)		
D	14 (2.5)	8 (1.4)	0 (0.0)	22 (3.9)		
SD	0 (0.0)	0 (0.0)	0 (0.0)	0 (0.0)		
	Dentists-interns: *p* = 0.013, Dentists-BDS: *p* = 0.012, Interns-BDS: *p* = 0.582					
D. Barriers						
Q22: Do you feel patient compliance and satisfaction require a dentist’s physical presence?						
SA	88 (15.9)	23 (4.2)	24 (4.3)	135 (24.4)	47.021	0.001
A	104 (18.8)	35 (0.0)	104 (18.8)	243 (37.6)		
N	41 (7.4)	22 (4.0)	59 (10.7)	122 (22.1)		
D	8 (1.4)	11 (2.0)	24 (4.3)	43 (7.7)		
SD	0 (0.0)	5 (0.9)	5 (0.9)	10 (1.8)		
	Dentists-interns: *p* = 0.214, Dentists-BDS: *p* < 0.001, Interns-BDS: *p* < 0.001					
Q23: Do you feel there is any fear of violating patient privacy by using teledentistry?						
SA	23 (4.2)	29 (5.2)	40 (7.2)	92 (16.6)	40.433	0.001
A	90 (16.3)	40 (7.2)	91 (16.5)	221 (40.0)		
N	63 (11.4)	17 (3.1)	82 (14.8)	162 (29.2)		
D	59 (10.7)	10 (1.8)	3 (0.5)	72 (13.0)		
SD	6 (1.1)	0 (0.0)	0 (0.0)	6 (1.1)		
	BDS-dentists: *p* < 0.001, BDS-interns: *p* < 0.001, Dentists-interns: *p* = 0.117					
Q24: Do you feel there is a low literacy level among the general population to comply with teledentistry?						
SA	71 (12.8)	24 (4.3)	65 (11.8)	151 (28.9)	3.238	0.198
A	106 (19.2)	48 (8.7)	114 (20.6)	268 (48.5)		
N	52 (9.4)	21 (3.8)	34 (6.1)	107 (19.8)		
D	12 (2.2)	3 (0.5)	3 (0.5)	18 (3.2)		
SD	0 (0.0)	0 (0.0)	0 (0.0)	0 (0.0)		
Q25: Do you feel there are high costs involved in teledentistry related infrastructure?						
SA	44 (8.0)	12 (2.2)	56 (10.1)	112 (20.3)	13.222	0.001
A	93 (16.8)	41 (7.4)	87 (15.7)	221 (36.9)		
N	75 (13.6)	24 (4.3)	64 (11.6)	163 (29.5)		
D	29 (5.2)	12 (2.2)	6 (1.1)	47 (8.5)		
SD	0 (0.0)	7 (1.3)	3 (0.5)	10 (1.8)		
	Interns-BDS: *p* = 0.210, Interns-dentists: *p* < 0.001, BDS- dentists: *p* < 0.007					
Q26: Do you feel time is required to upskill and apply the technology amongst oral care personnel?						
SA	64 (11.6)	21 (3.8)	50 (9.0)	135 (24.4)	10.211	0.006
A	112 (20.3)	37 (6.7)	132 (23.9)	281 (50.9)		
N	51 (9.2)	20 (3.6)	25 (4.5)	96 (17.3)		
D	14 (2.5)	18 (3.3)	9 (1.6)	41 (7.4)		
SD	0 (0.0)	0 (0.0)	0 (0.0)	0 (0.0)		
	Interns-BDS: *p* = 0.017, Interns-dentists: *p* < 0.001, BDS- dentists: *p* = 0.270					
Q27: Do you feel there are any chances inappropriate fees could be obtained from patients to justify teledentistry?						
SA	37 (6.7)	21 (3.8)	34 (6.1)	92 (16.6)	2.937	0.230
A	88 (15.9)	38 (6.9)	91 (16.5)	217 (39.2)		
N	80 (14.5)	23 (4.2)	67 (12.1)	170 (30.8)		
D	36 (6.5)	14 (2.5)	24 (4.3)	74 (11.3)		
SD	0 (0.0)	0 (0.0)	0 (0.0)	0 (0.0)		

*BDS*  Bachelor degree undergraduate students, *SA*  strongly agree, *A*  agree, *N*  neutral, *D*  disagree, *SD*  strongly disagree

As regards the attitudes towards TD, students’ responses reflected agreement towards the positive effect of TD on time (H = 87.543, *p* < 0.001, partial η² = 0.152, reflecting a large effect size), and costs reduction (H = 46.465, *p* < 0.001, partial η² = 0.077, reflecting a medium effect size). The perception of TD to monitor patients’ condition, indicated neutral tendency of the interns (H = 11.452, *p* < 0.001, partial η² = 0.014, reflecting a small effect size). The dentists showed a strong disagreement against the use of TD as a way to deliver oral health care (H = 46.321, *p* < 0.001, partial η² = 0.077, reflecting a medium effect size), and the use of videoconference for dental education (H = 12.172, *p* = 0.002, partial η² = 0.015, reflecting a small effect size), while the interns showed strong disagreement against TD effect on making dental examination easier compared to the students (H = 10.037, *p* = 0.007, partial η² = 0.011, reflecting a small effect size). All groups showed agreement towards TD improved accessibility of dental specialists to distant communities (*p* = 0.745).

The assessment of practice-related aspects revealed that undergraduate students significantly agreed on the necessity of patients to consents on sharing their records (H = 29.397, *p* = 0.004, partial η² = 0.046, reflecting a small effect size), and that TD could be used to support national oral health initiatives (H = 6.611, *p* = 0.037, partial η² = 4.745, reflecting a large effect size). Dentists reported neutral responses regarding the use of smartphone applications for patients’ (H = 18.565, *p* < 0.001, partial η² = 0.026, reflecting a small effect size). Dentists significantly expressed a positive agreement to the need for formal lectures or workshops to cover the gap in their knowledge about TD (H = 8.910, *p* = 0.012, partial η² = 8.927, reflecting a large effect size), however; they question that TD would be easy to use particularly compared to undergraduate students (H = 12.958, *p* = 0.002, partial η² = 0.024, reflecting a small effect size). On the other hand, a consistent agreement between the groups was detected as regards the need for hands-on training in using teledentistry (*p* = 0.353), and the use of intra-oral camera for dental examinations (*p* = 0.663).

For perceived barriers against the use of TD, dental students expressed greater worries regarding the physical presence of the dentists as a barrier against patient compliance and satisfaction (H = 47.021, *p* < 0.001, partial η² = 0.078, reflecting a medium effect size), and fear of violating patients’ privacy, compared to dentists and interns (H = 40.433, *p* < 0.001, partial η² = 0.066, reflecting a medium effect size). Dentists agreed more on the impact of infrastructure high costs (H = 13.222, *p* < 0.001, partial η² = 0.020, reflecting a small effect size), while interns raised their major concerns about the time required to upskill oral care personnel as a barrier against the use of TD (H = 10.211, *p* = 0.006, partial η² = 0.011, reflecting a small effect size). All groups agreed on that the low literacy level among the general population to comply with TD and charging fees from patients to justify TD related oral care service are considered barriers against the dissemination of TD application (at *p* = 0.198 and *p* = 0.230 respectively).

The responses of the groups following the educational presentation as regards their awareness about TD are presented in Table 4. The results revealed statistically significant improvement in TD awareness among participating dentists and dental students at *p* = 0.007 and *p* = 0.015 respectively. Contrary, dental interns showed no significant changes at *p* > 0.05. Dental students presented increased awareness by 60% points absolute change, corresponding to a 1.67% relative improvement compared to baseline. Dental interns showed improvement by 78% points absolute change, corresponding to a 4.11% relative improvement compared to baseline. Dentists presented enhanced awareness by 67% points absolute change, corresponding to a 3.05% relative improvement compared to baseline. Dental interns reported statistically significant agreement toward the usefulness of TD in dentistry compared to students, and significantly less neutral tendency compared to dentists (H = 13.744, *p* < 0.001, partial η² = 0.018, reflecting a small effect size).

**Table 4:** Participants’ responses regarding their knowledge towards Teledentistry following exposure to educational presentation

Variables	BDS *N* = 241 (%)	Interns *N* = 96 (%)	Dentists *N* = 216 (%)	Total *N* = 553 (%)	H	*p*-value
Knowledge						
Q1: Are you aware of Teledentistry?						
Yes	231 (41.8)	93 (16.8)	193 (34.9)	517 (93.5)		0.006
No	10 (1.8)	3 (0.5)	23 (4.2)	36 (6.5)		
Q2: Teledentistry is useful in all branches of dentistry?						
SA	61 (11.1)	26 (4.7)	32 (5.8)	119 (21.6)	13.744	0.001
A	87 (15.7)	51 (9.2)	89 (16.1)	227 (41.0)		
N	58 (10.5)	11 (2.0)	72 (13.0)	141 (25.0)		
D	29 (5.2)	8 (1.4)	17 (3.1)	54 (9.7)		
SD	6 (1.1)	0 (0.0)	6 (1.1)	12 (2.2)		
	Dentists-BDS: *p* = 0.086, Dentists-interns: *p* < 0.001, BDS-interns: *p* = 0.015					
Q3: Could current dental practices be accomplished through teledentistry?						
SA	49 (8.9)	22 (4.0)	50 (9.0)	121 (21.9)	5.488	0.065
A	92 (17.4)	49 (8.9)	89 (16.1)	230 (42.4)		
N	53 (9.6)	18 (3.3)	58 (10.5)	129 (22.9)		
D	35 (6.3)	7 (1.3)	15 (2.7)	57 (10.3)		
SD	8 (1.4)	0 (0.0)	4 (0.7)	12 (2.1)		
Q4: Can oral health care access be improved by teledentistry?						
SA	84 (15.2)	30 (5.4)	71 (12.8)	185 (33.4)	0.190	0.909
A	122 (22.1)	52 (9.4)	112 (20.3)	286 (51.8)		
N	25 (4.5)	14 (2.5)	33 (6.0)	72 (13.0)		
D	8 (1.4)	0 (0.0)	0 (0.0)	8 (1.4)		
SD	2 (0.4)	0 (0.0)	0 (0.0)	2 (0.4)		
Q5: Can teledentistry be useful for dental education and for upskilling health care workers through the internet?						
SA	102 (18.4)	40 (7.2)	70 (12.7)	212 (38.3)	2.579	0.275
A	112 (20.3)	47 (8.5)	128 (23.1)	287 (51.9)		
N	23 (4.2)	9 (1.6)	18 (3.3)	50 (9.1)		
D	2 (0.4)	0 (0.0)	0 (0.0)	2 (0.4)		
SD	2 (0.4)	0 (0.0)	0 (0.0)	2 (0.4)		
Q6: Can teledentistry be useful in diagnosis and management of oral diseases?						
SA	78 (14.1)	28 (5.1)	70 (12.7)	176 (31.9)	0.321	0.852
A	95 (17.2)	45 (8.1)	77 (13.9)	217 (39.2)		
N	40 (7.2)	12 (2.2)	38 (6.9)	90 (16.3)		
D	24 (4.3)	11 (2.0)	31 (5.6)	66 (11.9)		
SD	4 (0.7)	0 (0.0)	0 (0.0)	4 (0.7)		
Q7: Would teledentistry be helpful in consulting an expert about a patient’s problem effectively?						
SA	84 (15.2)	34 (6.1)	74 (13.4)	192 (34.7)	0.549	0.760
A	110 (19.9)	45 (8.1)	115 (20.8)	270 (48.8)		
N	39 (7.1)	13 (2.4)	18 (3.3)	70 (12.8)		
D	8 (1.4)	4 (0.7)	9 (1.6)	21 (3.7)		
SD	0 (0.0)	0 (0.0)	0 (0.0)	0 (0.0)		

*BDS* Bachelor degree undergraduate students, *SA* strongly agree, *A* agree, *N* neutral, *D* disagree, *SD* strongly disagree

## Discussion

TD represents an innovative transformation from telemedicine that relies on digital communication technologies to deliver oral healthcare services, facilitate remote consultations, and enhance patient-provider interactions. It includes a range of applications, including diagnosis, treatment planning, follow-up care, continuing dental education, and patient awareness programs. It can be associated with improved oral health outcomes and access to dental care via leveraging telecommunication technology, particularly in underserved populations and during situations where direct communication is limited as during pandemics [15]. It could be used to expand dental care access, reducing costs, and enhancing multidisciplinary collaboration [16].

A pre-validated, pre-tested self-administered questionnaire was used in the present study. The questionnaire consisted of the domains of knowledge, attitude, practice, and barriers. However, following the introduction of educational material on TD, only the knowledge section was repeated using the same survey link because that the main purpose was to assess the enhancement of awareness arising directly from the educational intervention. Following the COM-B Model for behavioral changes, the “capability” which relays on knowledge, is considered a fundamental prerequisite for change [16, 17]. Contrary, changes in attitudes, practices, and barrier perceptions would typically require extended time to be manifested since they depend on frequent exposure, repeated clinical application, and experience reinforcement [18]. Accordingly, the assessment of these domains was not applicable within the limitations of this study. However, restricting the post-educational intervention reassessment to knowledge provided focused and valid measurements of the enhanced awareness as compared to the baseline evaluation. Five-points Likert scale was used to assess the participants’ responses, as reported to be of acceptable validity and reliability [19].

The results of the present study revealed limited TD awareness particularly among interns and dental professionals (27.7%), where the dental students showed the highest focus of TD knowledge as attributed to higher chance of exposure to educational TD workshops or hands-on through the dental BDS program and their longer hours of internet usage (more than six hours daily) as evident in this study. Similar findings were reported by Alshammari and Almaktoom, who reported that up to 87.3% of dentists were unaware of or exposed for applying TD in their practice, and 60% questioned its usefulness for the improvement of dental health care [20]. Differences in reporting the degree of TD awareness could be attributed to differences in study populations regarding variations in clinical exposure, digital literacy or exposure to prior training, lectures or workshops on the clinical use of TD. It was also revealed that dentists with greater years of experience more than 10 years of experience, may possess limited TD knowledge for effective application in their practice [21].

The baseline disparity among the groups of the present study likely reflects differences in exposure. Current undergraduate students are accustomed to technology-driven learning, and hence, they are more open to adopting digital tools in dentistry. As indicated in this study, students’ responses revealed greater hours per day usage to the internet which could offers greater exposure to recent advances in health care delivery alternatives and TD applications tools. Interns who are in a transitional stage between education and independent practice may feel less prepared to use teledentistry without guidance. However, practitioners, who rely on established workflows raised concerns similar to those reported by previous studies regarding privacy, infrastructure, and ethical considerations [8, 20]. This could be attributed to dentists’ realities experience regarding infrastructure provision and adapting current systems to digital platforms [22].

Most of the participants in this study were neutral toward understanding the usefulness of TD in the field of dentistry, or its benefits for the current practice or its effectiveness in improving consulting medical cases and patients’ virtual consultations.

Dentists’ responses in this study revealed neutral tendency towards the positive impact of TD on oral health improvement of the recipient patients and its effectiveness to monitor dental patients’ condition, as could be attributed to the lower TD awareness rate in this study. High level of patient care can be delivered via TD particularly mitigating the risk of infectious diseases spreading as during COVID-19 associated with favorable patients’ acceptance [23, 24].

Undergraduate students and dentists reported positive impact regarding TD high usefulness in diagnosis, expert planning, and management of oral diseases. Additionally, undergraduates reflected their positive attitude relating to the effectiveness of TD in upskilling health care workers and dental education. As similarly reported by former studies, dental students reflected positive believes that TD could be effective to deliver readily accessible dental education [12, 25]. Contrary, dental professionals were more neutral regarding the TD effectiveness in delivering dental education, which could be linked to less digital skills of older age category (19.2% above the age of 40-year-old). As attributed to that, younger and “tech-savvy” participants would be more comfortable with using TD as they embrace using technologies and digital services more frequent than older dentists [26]. Furthermore, undergraduates presented higher leaning to the effect of TD in reducing time and cost needed to deliver dental care particularly being easier to reach distinct remote communities, similar to what reported by former studies [20, 27–29]. Lowered cost of offering dental care and monitoring patients’ condition via TD were also reported by dental students to be advantageous to encourage its wide usage [30].

Students’ perception to TD practice reflected clear understanding of patients’ consents to share their data and records for consultation using TD and the flipping tendency to replace traditional dental office with high quality intra-oral images and smart applications, particularly with the development of artificial intelligence systems that analyze dental images to improve remote diagnostics [31, 32]. This concept was found difficult to be accepted by dentists in this study as attributed to the tendency to retain the same traditional in-office visits, their concerns about the level of care delivered through TD and being hesitant to TD training needs [27, 28]. Dental professionals and interns participated in this study reported that effective implementation of TD in dental practice, could be achieved via the provision of specialized training workshops and specialized TD courses. Cohort findings were concluded by former studies that adequate TD awareness would broaden its usage and application [33, 34].

All participants in this study agreed on that low literacy of patients and justification of charging fees could be an impediment factors against effective use of TD. Patients with low oral hygiene literacy knowledge and limited literacy level could represent a key barrier against effective communication [35]. In addition, the lack of familiarity with TD, the necessary digital literacy, and patient acceptance of virtual consultation, could be among the TD challenges that require careful consideration [36].

Dental professionals expressed greater skepticism specifically regarding violating patients’ privacy, the urgent need to upskill dental healthcare providers for the effective use of TD across different branches, and the importance of upgrading costly infrastructure and developing policies to effectively integrate TD into oral health care services. As reported by Chaudhary et al., that despite moderate rate of TD awareness among different categories of dental professional, the limited infrastructures facilities, set-up cost and restricted information technology are considered among the main challenges to be overcomed by strategic governmental initiatives [37]. In addition, other studies also reported that poor internet connection, and absence of legal regulation of TD would cause serious impediment to effective usage of TD [38, 39]. Impediments against TD integration within the oral health systems in form of a business model and cost-benefit needs are among the TD barriers to active implementation particularly in developing countries [40].

Following the introduction of educational intervention, clear improvement in awareness across all groups was evident. Dental interns did not show a statistically significant improvement in awareness as influenced by limited sample size (*N* = 96) compared to students and dentists. Participants expressed uniform agreement on TD usefulness for dental education and upskilling health care providers parallel to its applicability in diagnosis and management of oral diseases via facilitating experts’ consultation. This positive outcome supports earlier studies recommending structured teaching to improve attitudes toward TD [9, 41, 42]. Didactic TD courses would encourage students’ understanding and acceptance, underscoring the role of curricular integration [12]. Enhanced TD awareness of students and dental professionals would promote its utilization in their carrier [10, 20]. Directed campaign to educate dentists as well as to the public about TD technology and its potentials in facilitating access to oral and dental care, is highly recommended to strengthen its fundamental application.

Enhancing the awareness of TD among dental students, interns and professionals is considered essential for maximizing its benefits in clinical practice and public health. Understanding the scope of TD in terms of remote diagnosis, consultation, patient education and interdisciplinary collaboration, enables future and current practitioners to integrate these tools effectively into patient care. It also equips dental providers to adapt to evolving healthcare models, and remain competent to changes in their community needs. From a clinical and public health perspective, remote dental care has the potential to enhance access to oral healthcare, especially in underserved areas. Its role has been particularly emphasized in the post-COVID-19 context, where remote consultation and monitoring became essential [6, 8].

This study is among the few to evaluate the knowledge, attitudes, and perceived barriers to TD using a structured, pretested, and pre-validated questionnaire to assess different levels of dental training and practice across professional strata (undergraduate students, dental interns, and dentists). However, among the limitations of this study that its cross-sectional design impedes causal inference and the reliance on self-reported data may introduce a form of bias. This study was conducted at a single institution located in the city of Cairo, thus limiting the generalization of its findings. The study may also be subjected to selection bias through using a convenience sampling via WhatsApp groups and the dental syndicate. Furthermore, the absence of a control group not exposed to the educational intervention may restrict the attribution of detected changes solely to the intervention, however; the first assessment was used as the baseline control to compare the findings with the re-assessment outcomes. In addition, only the knowledge domain was reassessed following the educational intervention, since it could not estimate time dependent long-term changes in the other domains. The immediate re-assessment following the educational intervention may introduce testing bias via exposing the participants to the same questionnaire with short interval, as well as Priming bias since the responses could be influenced by increased topic awareness resulting in overestimation of the recorded imorovement in TD knowledge. This study didn’t interpret the TD awareness and acceptance to theoretical framework as the Technology Acceptance Model, which may restrict the contextualization of the study findings [17].

Accordingly, future researchers should adopt longitudinal study designs to assess the sustained changes of enhanced teledentistry awareness in attitude, clinical practice, and barriers perception with further exploration of patient satisfaction and cost-effectiveness of TD in healthcare delivery systems. Furthermore, running multi-center studies and comparing different educational interventions as interactive lectures and workshops, could point to the most effective approaches to enhance TD awareness among dental professionals, particularly among larger interns’ cohorts.

## Conclusions

 Under the limitations of the present study, the following can be concluded:

1. The baseline awareness of teledentistry among different categories of dental professionals was limited, particularly among interns and dentists, while greater knowledge was positively perceived by undergraduate students, which underscores the need to integrate Teledentistry into undergraduate and continuing education curricula to adopt digital health care into dental practice.
2. Patient’s privacy, cost-effectiveness, and practical applicability of teledentistry were among the main concerns particularly expressed by the dentist, thus highlighting the importance of developing regularity policies, ethical guidelines, and training on operational legal aspects to facilitate confident implementation in dental practice.
3. Educational intervention positively improved teledentistry awareness, highlighting the value of structured teaching to support the adoption of targeted educational programs, and continuing professional development initiatives.

## Supplementary Information

Supplementary Material 1.

## Data Availability

The datasets used and/or analysed during the current study are available from the corresponding author on reasonable request.
