# Supplementary material for: Dental professionals’ knowledge and awareness of teledentistry
Source: BMC Oral Health. 2026 Jun 3;26:1058. doi: 10.1186/s12903-026-08717-5 (PMC13270566; doi:10.1186/s12903-026-08717-5)
Supplement: Supplementary file 1 — Supplementary Material 1. [file 12903_2026_8717_MOESM1_ESM.pdf]

Dental Professionals' Knowledge and Awareness of Teledentistry Questionnair

Section 1: Personal Information

1. Age Category
a. 18-24
b. 25-29
c. 30-40
d. 40-50
e. 50-60
f. 60+

2. Gender Male or Female

3. Educational level:
a. Undergraduate Student
    - Year 1
    - Year 2
    - Year 3
    - Year 4
    - Year 5
b. Postgraduate Candidate
    - M.D
    - M.S
c. Intern
d. Dentist

If dentist, then a special section opens for them

4. Working in:
    a. Governmental sector
    b. Private sector
    c. Both

5. Your Specialty:
    a. Basic Oral Sciences (Academic)
    b. General dentist
    c. Endodontist and Restorative dentist
    d. Prosthodontist
    e. Pediatric dentist
    f. Periodontist
    g. Orthodontist
    h. Oral and Maxillofacial surgeon
    i. Oral and Maxillofacial radiologist

6. Years of experience:
a.<5 years
B. 5-10 years

c. >10 years

7.Frequency of Internet usage per Day

a. <3 hours
b. 3-6 hours
c. >6 hours

Section 2:

A. Knowledge: (Yes or No)

1. Are you aware of teledentistry?

2. Do you feel teledentistry is useful in all branches of dentistry?

3. Do you feel current dental practices could be accomplished through teledentistry?

4. Can oral health care access can be improved by teledentistry.

5. Do you feel teledentistry is useful for dental education and upskilling health care workers over the internet?

6. Do you feel teledentistry is useful in diagnosis and management of oral diseases?

7. Do you feel teledentistry would help in consulting an expert about a patient's problem effectively?

B. Attitude: (5-point-Likert)

8. Do you feel you can monitor the patient’s condition?

9. Do you feel teledentistry could be a way of oral health care delivery?

10. Do you feel teledentistry could make dental examination easier?

11. Do you think that teledentistry saves time for the dentist?

12. Do you feel teledentistry could reduce the cost of dental services?

13. Do you feel videoconference (a medium of teledentistry) could be used to educate dental students effectively?

14. Do you feel teledentistry could improve accessibility of dental specialists to distant communities for their oral needs?

C. Practice (5-point-Likert)

15. Do you feel patients would provide consent to share their dental reports through teledentistry to another dentist?

16. Do you feel usage of teledentistry would support an initiative for delivering oral health to everyone on a national level?

17. Do you think that dental examinations are accurate via computers and intraoral camera as in the traditional office setting?

18. Does teledentistry include usage of smartphone applications for patient consultation?

19. Do you feel teledentistry is easy to use?

20. Do you feel hands-on training is required in using teledentistry?

21. Do you feel a lecture/course is required to learn about teledentistry?

D. Barriers (5-point-Likert)

22. Do you feel patient compliance and satisfaction require a dentist's physical presence?

23. Do you feel there is any fear of violating patient privacy by using teledentistry?

24. Do you feel there is a low literacy level among the general population to comply with teledentistry?

25. Do you feel there are high costs involved in teledentistry related infrastructure?

26. Do you feel time is required to upskill and apply the technology amongst oral care personnel?

27. Do you feel there are any chances inappropriate fees could be obtained from patients to justify teledentistry?
